# Inflammatory Response to TiO_2_ and Carbonaceous Particles Scales Best with BET Surface Area

**DOI:** 10.1289/ehp.115-a290b

**Published:** 2007-06

**Authors:** Tobias Stoeger, Otmar Schmid, Shinji Takenaka, Holger Schulz

**Affiliations:** GSF - National Research Center for Environment and Health, Institute of Inhalation Biology, Neuherberg/Munich, Germany, E-mail: tobias.stoeger@gsf.de

In an attempt to identify the proper dose metric for particle toxicity, Wittmaack (2007) reanalyzed our dose–response data (Stoeger et al. 2006) and that of Oberdörster et al. (2005) on acute lung inflammation in rodents after instillation of various particle types. Out of particle BET surface area (*S_BET_*), particle number, joint length, and “geometric” surface area, Wittmaack concluded that particle number tends “to work best” as dose metric. We disagree with his conclusion.

First, we wonder why Wittmaack (2007) used our data but ignored the data of Oberdörster et al. (2005) for the identification of the best dose metric. Figure 1 shows our dose–response data (in mice) for six different types of ultrafine carbonaceous particles (10–50 nm) and the data of Oberdörster et al. (2005) for fine (~ 250 nm) and ultrafine (~ 20 nm) TiO_2_ particles; we present the data for rats, which was reanalyzed by Wittmaack, and also the mouse data from Oberdörster et al. (2005). In Figure 1 the inflammatory response after 24 hr is expressed as the ratio of the polymorphonuclear leukocytes (PMNs) to lavaged cells, and the instilled dose is normalized to lung weight, because this facilitates interspecies comparison (Oberdörster et al. 2005). As suggested by Wittmaack (2007), we limit our discussion to the linear response regime [analogous to his Figure 3 (Wittmaack 2007)]. For this data set, the linear correlation coefficient *R*^2^ is 0.46, 0.51, 0.67, and 0.72 for particle number, joint length, “geometric” surface area, and *S_BET_*, respectively. Particularly, the response to the fine particles, as represented by the red fit line (almost identical to the *y*-axis in Figure 1A), is not adequately described by particle number (Figure 1A), whereas *S_BET_* works well for all particle sizes (Figure 1B). Although we do not suggest *S_BET_* as a “universal” dose metric (chemistry, charge, etc., are also relevant), we conclude that for the dose metric examined here, *S_BET_* is the most relevant dose parameter. Wittmaack’s preference for particle number appears to be the result of an unsubstantiated restriction of his analysis to our data, which is dominated by particles in a relatively narrow size regime between about 10 and 25 nm.

Second, all investigated dose parameters (except *S_BET_*) depend on accurate determination of the mean particle diameter, <*d*>, requiring tedious and potentially uncertain single particle analysis. Wittmaack (2007) acknowledged potentially large errors in <*d*> for particles below about 20 nm [i.e., for four out of our six (carbonaceous) particle types]. Being aware of these limitations, we intentionally reported only a range of observed particle diameters (not <*d*>) in our article (Stoeger et al. 2006). Unfortunately, Wittmaack did not discuss his conclusions in light of these methodologic limitations. Especially for the smallest particle type (here spark-generated carbon particles with <*d*> = 9.8 nm), preferential particle selection is likely to result in an overestimation of <*d*>. Assuming a 25% sizing error, this yields a systematic error of + 100% in particle number (~ <*d*>^−3^), which shifts these data points far away from the linear fit line (see error bars in Figure 1A). In contrast, *S_BET_* requires only a single measurement on an aliquot of the administered particles; that is, it is not adversely affected by problems associated with single particle analysis, and it adequately accounts for potentially important particle characteristics such as particle morphology and surface porosity.

In summary, we do not agree with the dose–response interpretation of our data by Wittmaack (2007). We conclude that *S_BET_* (and not particle number) is the best dose parameter, accounting for 72% (*R*^2^ = 0.72) of the observed inflammatory response for both data sets spanning a size range of 10–250 nm.

## Figures and Tables

**Figure 1:**
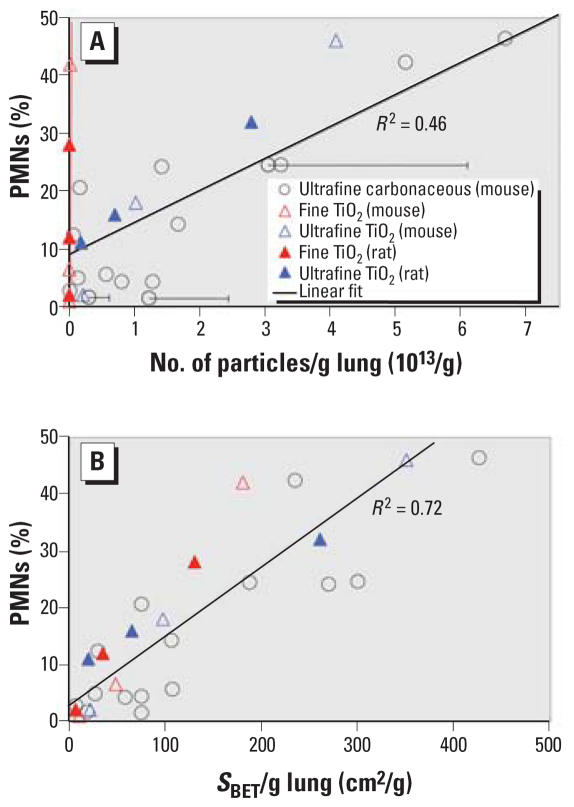
Acute pulmonary inflammatory response (PMNs) to TiO_2_ [Oberdörster et al. 2005; Figure 4 and Figure S-2 (Supplemental Material available online at http://ehp.niehs.nih.gov/members/2005/7339/supplemental.pdf)] and carbonaceous particles (Stoeger et al. 2006; Figure 1) in rats and mice, with particle number (*A*) and *S_BET_* (*B*) as the dose metric.
